# MicroRNAs: New Regulators of Toll-Like Receptor Signalling Pathways

**DOI:** 10.1155/2014/945169

**Published:** 2014-03-20

**Authors:** Xiaobing He, Zhizhong Jing, Guofeng Cheng

**Affiliations:** ^1^State Key Laboratory of Veterinary Etiological Biology, Key Laboratory of Veterinary Public Health of Ministry of Agriculture, Lanzhou Veterinary Research Institute, Chinese Academy of Agricultural Sciences, Lanzhou 730046, China; ^2^Key Laboratory of Animal Parasitology, Ministry of Agriculture, Shanghai Veterinary Research Institute, Chinese Academy of Agricultural Sciences, Shanghai 200241, China

## Abstract

Toll-like receptors (TLRs), a critical family of pattern recognition receptors (PRRs), are responsible for the innate immune responses via signalling pathways to provide effective host defence against pathogen infections. However, TLR-signalling pathways are also likely to stringently regulate tissue maintenance and homeostasis by elaborate modulatory mechanisms. MicroRNAs (miRNAs) have emerged as key regulators and as an essential part of the networks involved in regulating TLR-signalling pathways. In this review, we highlight our understanding of the regulation of miRNA expression profiles by TLR-signalling pathways and the regulation of TLR-signalling pathways by miRNAs. We focus on the roles of miRNAs in regulating TLR-signalling pathways by targeting multiple molecules, including TLRs themselves, their associated signalling proteins and regulatory molecules, and transcription factors and functional cytokines induced by them, at multiple levels.

## 1. Introduction

Toll-like receptors (TLRs), an important family of pattern recognition receptors (PRRs), are responsible for the recognition of pathogen-associated molecular patterns from infectious pathogens. This recognition triggers the production of large amounts of inflammatory cytokines, type I interferons (IFNs), and antiviral proteins through the activation of interferon regulatory factor (IRF) 3, IRF7, activator protein-1 (AP-1), and nuclear factor-kappa B (NF-*κ*B) [1–3]. In addition, the TLR-signalling pathways are strictly and finely regulated by positive or negative modulation at multiple levels to prevent excessive inflammation and achieve a balanced output [2, 4, 5]. Several mechanisms are responsible for the regulation of the TLR-signalling pathways. These include physical interactions, conformational changes, phosphorylation, ubiquitylation, and proteasome-mediated degradation involving various regulatory molecules [2, 4, 5]. Among the many regulatory molecules, microRNAs (miRNAs) have received considerable attention as a newly identified family of regulators involved in fine-tuning the TLR-signalling pathways [6–12].

**Figure 1:**
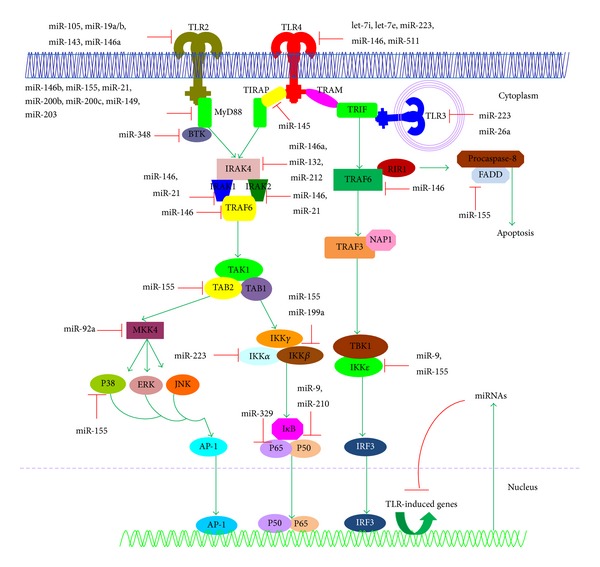
The fine-tuning of the TLR-signalling pathways by miRNAs. An array of miRNAs are involved in regulating of the TLR-signalling pathways and innate immune responses by targeting multiple molecules at multiple levels, including TLRs themselves, TLR-associated signalling proteins, TLR-associated regulatory molecules, TLR-induced transcription factors, and TLR-induced functional cytokines.

miRNAs are a class of small noncoding RNAs (about 22 nucleotides in length) that regulate gene expression by binding to the 3′-untranslated regions (UTRs) of target messenger RNAs (mRNAs), typically resulting in protein translation repression or mRNA degradation [13–15]. miRNAs are involved into many biological processes, including development, differentiation, growth, homeostasis, stress responses, apoptosis, and immune activation, in organisms such as animals, plants, and even some DNA viruses [13–15]. To date, more than 30,000 miRNAs have been identified in at least 206 species. One prediction is that up to 30% of all human genes are regulated by miRNAs in many cell types, including immune and epithelial cells [13–15]. Therefore, miRNAs may serve as important regulators for controlling the differentiation of immune cells as well as the immune responses to pathogen infections [16–18]. Recent studies have indicated that miRNAs play important roles in regulating the TLR-signalling pathways and innate immune responses and function as immunomodulators for the complex regulatory networks [6–12]. Remarkably, many molecules that are involved in the TLR-signalling pathways (including signalling proteins, regulatory molecules, transcription factors, cytokines, and TLRs) are regulated by an array of miRNAs (Figure 1) [6–12].

Here, we review the recent findings regarding the relationship between miRNAs and the TLR-signalling pathways. Overall, we summarize the current understanding of the mechanisms of TLR-signalling pathways regulated by miRNAs. Then, we focus on the roles of miRNAs in regulating the TLR-signalling pathways by targeting TLRs themselves, their associated signalling proteins and regulatory molecules, and transcription factors and functional cytokines induced by them.

## 2. Regulation of miRNA Expression by the TLR-Signalling Pathways

Many studies have demonstrated that miRNAs expression profiles are subject to change in different cell lines when stimulated by the ligands in the TLR-signalling pathways. Briefly, in 2006, Baltimore Lab first documented that the upregulated expression of miR-146a, miR-155, and miR-132 in human monocytes is related to stimulation with the lipopolysaccharide (LPS) [19]. Subsequent studies found that miR-223, miR-147, miR-9, miR-27b, and let-7e are induced by stimuli from other TLRs, by pathogen infection, and by IL-1*β* [52, 57–59]. Furthermore, several miRNAs such as miR-155, miR-146, and miR-21 are able to target some molecules involved in the TLR-signalling pathways, although the expression of some of these miRNAs depends on the stimulation by TLR ligands [6, 8, 20, 21]. It is also noted that miRNAs may also induced in a temporal-specific manner. For example, miR-146 and miR-155 are highly expressed within 2 h after LPS treatment and are thus early-response miRNAs, whereas miR-21 is expressed in macrophages at a later time after LPS treatment and is thus a late-response miRNA [6, 8, 9, 19–21, 63]. The different expression of these miRNAs in TLR-induced cells may also be attributed to the duration of treatment, the technology used, and the different cell types. Nonetheless, these studies clearly indicate that molecules involved in the TLR-signalling pathways can regulate miRNA expression (Table 1).

To date, almost all of miRNA expressions appearto depend on the TLR-induced NF-*κ*B and MAPK pathways (Table 1). In 2006, miR-146a was the first reported miRNA whose expression depends on the NF-*κ*B pathway in human THP-1 monocytes after LPS stimulation [19]. Since then, many studies have further identified subsets of miRNAs related to the TLR-induced NF-*κ*B-dependent pathway. The expression of many miRNAs including miR-146a, miR-155, miR-132, miR-223, miR-147, miR-9, miR-27b, let-7e, miR-21, miR-16, miR-23b, miR-30b, miR-301a, and miR-125b is induced in an NF-*κ*B-dependent manner after TLR stimulus or pathogen infection [10, 19–23, 36–39, 49, 50, 53, 57–64, 70]. For example, miR-9 expression is directly induced by LPS via the TLR4-MyD88-NF-*κ*B-dependent pathway in human monocytes and neutrophils [58]. In addition, miR-155 expression is induced in the NF-*κ*B-dependent manner in various cell types after many stimuli, including LPS and LMP1, the viral latent protein of the Epstein-Barr virus (EBV) [36, 37]. miR-146a expression can also be induced through the NF-*κ*B-dependent pathway in response to various immune mediators, such as LPS, IL-1*β*, LMP1, and tumour necrosis factor- (TNF-) *α* [19, 22, 23, 39]. Conversely, some miRNAs (miR-29b, let-7i, miR-98, miR-107, miR-27a, and miR-532-5p) are downregulated by the TLR-induced NF-*κ*B-dependent pathway [39, 72–77, 120]. It is necessary to point out that miR-125b expression related to the TLR-activated NF-*κ*B-dependent pathway remains controversial and needs to be further investigated in the future [40].

The MAPK pathway is also involved in regulating miRNA expression (Table 1). For example, miR-21, miR-146b, miR-155, and miR-146b-5p are reportedly upregulated through the heterodimers Fos and Jun in different cell types in response to various stimuli [36, 41, 65, 66, 71, 121]. In contrast, the MAPK pathway is also involved in the downregulation of miRNA expression, for example, miR-99a [24]. However, other cellular pathways are also responsible for regulating miRNA expression. For example, the expression of miR-132 is regulated by the cyclic AMP response element-binding protein and the transcriptional coactivator p300 [50]. Expression of miR-143/145 cluster is downregulated by the Janus kinase 1 (JAK1)—signal transducer and activator of transcription 1 (STAT1)—dependent pathway [85].

Since the expressions of most TLR-responsive miRNAs are related to the corresponding ligand stimulation or the infection of some pathogens, it is reasonable to conclude that these miRNAs may be not only involved in regulating the innate immune responses, ensuring host protection, but also play important roles in the pathogenesis of some infectious diseases. Because TLR and miRNA expression profiles are limited to certain cell types, the different TLR distributions in different immune cells might also have different miRNA expressions. Currently, our understanding of the TLR-induced miRNAs has significantly expanded, with breakthroughs providing insights into the finely tuned miRNA-mediated regulation of the TLR-signalling pathways.

## 3. miRNA-Mediated Regulatory TLR-Signalling Pathways 

miRNAs regulate TLR-signalling pathways at several layers, including regulation of TLR expression, TLR-associated signalling proteins and regulatory molecules, and TLR-induced transcription factors and functional cytokines (Figure 1).

### 3.1. miRNAs-Mediated Regulatory TLR Expression

It is well recognized that the activation of TLR-signalling pathways is required for hosts to eliminate invading pathogens. However, excessive activation of these pathways may also disrupt immune homeostasis, leading to some diseases such as autoimmune diseases, chronic inflammatory diseases, or cancer [2, 4, 5]. Therefore, precise regulation of TLR-signalling pathways is especially important [6–12]. Since miRNAs act as a class of key regulators of gene expression, the regulation of TLR expression may be one of the effective points at which miRNAs target TLRs (Table 2).

To date, several miRNAs have been shown to regulate TLR expression. Among them, the let-7 miRNA family, including let-7e and let-7i, can regulate TLR4 expression. Overexpression of let-7e by miRNA mimics resulted in the downregulation of TLR4 expression in mouse peritoneal macrophages, and inhibition of let-7e by antisense miRNA led to the upregulation of TLR4 expression [62]. Let-7i regulates TLR4 expression in human biliary epithelial cells [72]. This different member of the let-7 miRNA family is found in macrophages and epithelial cells, where it regulates TLR4 expression, probably due to the differences in the TLR-induced miRNA expression profiles of different cell types. Another TLR-induced miRNA, the myeloid-specific miR-223, can regulate both TLR4 and TLR3 expression in granulocytes [53]. A recent study found that miR-146a can also negatively regulate TLR4, resulting in accumulation of oxidized low-density lipoprotein (oxLDL) accumulation and an inflammatory response in macrophages [25]. In addition, miR-511 functions as a putative positive regulator of TLR4 under cell cycle arrest conditions, whereas it seems to inhibit TLR4 expression under similar conditions in monocytes and dendritic cells (DCs) [78]. Moreover, miR-26a can negatively regulate the TLR3 signalling pathway by targeting TLR3 expression in rat macrophages and ameliorates pristane induced arthritis in rats [80].

TLR2 is another receptor regulated by miRNAs (Table 2). The expression of TLR2 is negatively regulated by miR-146a and miR-105, respectively [81, 82]. In addition, miR-19a/b upregulates TLR2 expression in fibroblast-like synoviocytes of rheumatoid arthritis patients [83]. Overexpression of miR-19a/b by miRNA mimics not only reduces TLR2 protein expression but also significantly inhibits the activities of the TLR2-triggered cytokines and kinases [83]. Furthermore, miR-143 can inhibit the expression of TLR2, leading to the suppression of the invasion and migration of a subset of human colorectal carcinoma cells [84].

Overall, these studies suggest that miRNAs play an important role in the constitutive expression of TLRs. Further mining of other TLRs regulated by miRNAs is still needed. Nevertheless, miRNA regulatory TLR downstream signalling molecules and/or transcription factors seem more effective than miRNAs directly targeting TLRs to abolish receptor expression and thus completely shut down the TLR-signalling pathway.

### 3.2. miRNA-Mediated Regulatory TLR-Associated Signalling Proteins

TLRs recruit many types of proteins for these signalling pathways upon ligand binding. These proteins include adaptor molecules [myeloid differentiation factor 88 (MyD88), Toll/Interleukin-1 receptor (TIR) domain-containing adapter molecule (TRIF), TIR domain-containing adaptor protein (TIRAP), and TRIF-related adaptor molecule (TRAM)], various kinases [IL-1R-associated kinases (IRAKs), Bruton's tyrosine kinase (BTK), MAPK kinases (MKKs), TAK1-binding proteins (TABs), and I*κ*B kinases (IKKs)], and ubiquitin ligases [TNFR-associated factors (TRAFs)]. Recently, these molecules have also been shown to be targeted by a set of miRNAs, especially the TLR-induced miRNAs [1–12] (Table 2).

Among these miRNAs identified, miR-146a, one of key TLR-induced miRNAs, inhibits the TLR-signalling pathway by targeting IRAK1 kinase and TRAF6 ligase [19, 26–28, 85]. IRAK1 and TRAF6 are the important components of the MyD88-dependent pathway for activating most TLRs-mediated signalling pathways (except TLR3) in many cell types, including the THP-1 cell line [9, 19, 26–28]. Recently, IRAK2, another IL-1R-associated kinase, has also been shown to be regulated by miR-146a, although its relevance in the TLR-signalling pathways remains to be further determined [19, 26]. In addition, miR-146a can inhibit IL-8 expression, suggesting that this negative regulation might be an important mechanism of severe inflammation during the innate immune response [22]. Moreover, miR-146b, another miRNA of the miR-146 family, is an IL-10-responsive miRNA with anti-inflammatory activity [79]. miR-146b can modulate the TLR4-signalling pathway by directly targeting multiple proteins, including TLR4, MyD88, IRAK1, and TRAF6 [79]. This modulation leads to a significant reduction of several inflammatory cytokines and chemokines. The third IL-1R-associated kinase, IRAK4, has been shown to be targeted by miR-132, miR-212, and miR-146a, decreasing the production of inflammatory cytokines [51]. A recent study demonstrated that miR-29 as tumor suppressor miRNA is one of the negative regulators of TRAF4 expression in metastatic prostate cancer [89].

miR-155, another important TLR-induced miRNA, can target some of several signalling proteins in TLR-signalling pathways. These proteins mainly include some of the components of the NF-*κ*B pathway, such as Fas-associated death domain protein, IKK*β*, IKK*ε*, and the receptor- (TNFR superfamily) interacting serine-threonine kinase 1 [38, 40]. Next, miR-155 has been shown to inhibit the p38 MAPK signalling pathway and inflammatory cytokine production in human DCs in response to microbial stimuli [38]. Furthermore, miR-155 can regulate TAB2 (a signalling molecule downstream of TRAF6) in human monocyte-derived DCs [38]. Recently, MyD88 has been shown to be regulated by miR-155, miR-149, and miR-203 [29, 30, 86, 87]. In addition, TIRAP (also known as MAL), another MyD88 adaptor-like protein that acts as a bridging adaptor for TLR2- and TLR4-mediated MyD88-dependent signalling pathways, has been identified as a target of miR-145 in hematopoietic stem/progenitor cells [31].

In addition to previously described miRNAs, the miR-200 family (such as miR-200b and miR-200c) can also regulate the expression of MyD88. This regulation can modify the efficiency of the TLR4-signalling pathway and thus affect host innate defences against microbial pathogens [88]. miR-21 also inhibits the expression of MyD88 and IRAK1, leading to the upregulation of the JNK/c-Jun signalling pathway, the ERK/c-Fos pathway, and signalling by type I IFNs during RNA virus infection [54]. In addition, miR-346 can target Bruton's tyrosine kinase (BTK), a critical tyrosine kinase involved in the TLR4, TLR7, TLR8, and TLR9 signalling pathways for NF-*κ*B activation [69, 90]. Next, miR-223 has been shown to target IKK*α* (one of the serine-threonine kinases in the canonical NF-*κ*B pathway) in human monocytes/macrophages [91]. miR-199a can target IKK*β* (another serine-threonine kinase) in human ovarian cancer cells and/or endometrial stromal cells, leading to suppression of the NF-*κ*B pathway activation and reduced IL-8 production [122, 123]. Moreover, miR-92a can target mitogen-activated protein kinase kinase 4 and thus inhibit the TLR4-triggered inflammatory response in macrophages [92].

Collectively, some of the key components of TLRs-associated signalling proteins are regulated by certain miRNAs, although relatively few signalling proteins have been identified so far. These studies suggest that miRNA could result in timely and appropriate toning down and/or termination of the TLR-signalling pathway by targeting critical signalling proteins, once a TLR is triggered.

### 3.3. miRNA-Mediated Regulatory TLR-Induced Transcription Factors

Activation of certain transcription factors, such as IRF, NF-*κ*B, AP-1, and STAT, is a key functional step for TLR-signalling pathways. Theoretically, targeting transcription factors by miRNAs may globally affect TLR-induced gene expressions. Many studies have experimentally demonstrated that miRNA also plays a vital role in regulation of TLR-induced transcription factors [124, 125]. As discussed above, miRNAs induced by certain TLRs-activated transcription factor-dependent signalling pathways usually provide feedback to regulate their activation. Many studies reveal that miRNAs directly and/or indirectly regulate the expressions of TLR-activated transcription factors [6–12] (Table 2). Generally, NF-*κ*B is considered the most important transcription factor in TLR-induced signalling pathways. It has been demonstrated that miR-9 (one of the TLR-responsive miRNAs) directly targets NF-*κ*B1 mRNA [58]. Because the TLR4 agonist LPS induces miR-9 expression in a MyD88- and NF-*κ*B-dependent pathway manner, miR-9-mediated feedback may control the TLR-signalling pathways by fine-tuning NF-*κ*B1 expression [58]. In addition, a recent study has also demonstrated that miR-210 can target NF-*κ*B1 under induction by LPS in murine macrophages [93]. Furthermore, miR-329 plays a pivotal role in the inhibition of IL-6 mRNA expression by targeting the NF-*κ*Bp65 [94].

miRNA can also target other TLR-activated transcription factors. For example, miR-17-5p and miR-20a in myeloid-derived suppressor cells can target STAT3 and thus alleviate the suppressive function of myeloid-derived suppressor cells [95]. In addition, miR-223 has also been shown to target STAT3, resulting in the inhibition of the proinflammatory cytokines IL-6 and IL-1*β* production in macrophages [55]. Recently, a transcriptional corepressor CCAAT/enhancer binding protein-*β* has been identified as a target of miR-155. The effect of miR-155 leads to decreasing the expression levels of granulocyte colony-stimulating factor and possibly IL-6 in splenocytes [32, 33]. Another transcriptional coactivator, p300, which often associates with the cAMP-responsive element-binding protein, is targeted by miR-132 in lymphatic endothelial cells infected with Kaposi's sarcoma-associated herpesvirus [50]. Furthermore, Forkhead box p3 (a transcription factor required for the regulatory T cells) and E26 transformation-specific sequence 2 have also been identified as targets of miR-155 [34, 96]. Interestingly, when induced by the NF-*κ*B-dependent pathway, miR-27b directly targets peroxisome proliferator-activated receptor *γ* and inhibits LPS-induced TNF secretion after LPS treatment [59].

### 3.4. miRNA-Mediated Regulatory TLR-Induced Cytokines

Activation of TLR signalling through recognition of pathogen-associated molecular patterns leads to the transcriptional activation of genes encoding for proinflammatory cytokines, chemokines, and costimulatory molecules. These cytokines play an important role in eradicating infectious pathogens and recruiting inflammatory cells to the infection site for effective host defence. Several key TLR-induced functional cytokines such as type I IFNs, TNF, IL-6, IL-12, and IL-10 have been demonstrated to be regulated by miRNAs. Bioinformatics analysis also indicated that the mRNAs encoding these cytokines and chemokines have the binding sites for miRNAs [126–128] (Table 2).

A recent study demonstrated that miR-146a sequentially suppresses the production of type I IFNs, TNF, IL-1*β*, and IL-6 by targeting IRAK1, IRAK2, and TRAF6 in macrophages during vesicular stomatitis virus (VSV) infection or during LPS tolerance [26–28]. In addition, miR-466l can directly bind to the 3′-UTR of IFN-*α* and thus reduce IFN-*α* expression during VSV and EBV infections [98]. Moreover, miR-26a, miR-34a, miR-145, and let-7b directly regulate the expression of IFN-*β* by targeting the IFN-*β* 3′-UTR [11, 99]. On the other hand, certain cytokines such as type I IFNs can also affect miRNA expression. For example, the activation of IFN-*α* can suppress two abundantly expressed miRNAs, miR-378 and miR-30e. This suppression allows release of cytolytic mRNAs, resulting in augmented natural killer cell cytotoxicity [129]. Furthermore, miRNAs can affect antiviral immunity through modulating IFN downstream signalling. For example, owing to the absence of a miR-29a cluster in the thymic epithelium, high expression of IFN-*α* receptor in the thymic epithelium triggers suboptimal signalling and then results in a rapid loss of thymic cellularity [130]. In addition, miR-132 has been shown to perform a negative effect on the expression of interferon-stimulated genes, facilitating viral replication [50].

Aside from type I IFNs, TNF mRNA contains a binding site that can be targeted by miR-125b in mouse RAW 264.7 macrophages [40]. Recently, it has been demonstrated that miR-187 directly targets TNF-*α* mRNA and indirectly decreases IL-6 and IL-12p40 expression via downmodulation of I*κ*B*ζ*, a master regulator of the transcription of these latter two cytokines [100]. Next, it has been shown that IL-6 can be targeted by several miRNAs such as miR-16, miR-365, and miR-142-3p, subsequently reducing the endotoxin-induced mortality by restricting TLR signalling through a feedback mechanism [11, 103, 104]. In addition, bioinformatics analysis indicates that the 3′-UTR of the IL-6mRNA contains a let-7-binding site. However, many let-7 family miRNAs can usually be suppressed after TLR stimulation, suggesting that let-7 may contribute to the expression of IL-6 [73]. But, the relationship between the let-7 family and IL-6 remains to be experimentally determined. Another study has shown that IL-10 is also regulated by miR-106a and miR-106b in human Burkitt's lymphoma Raji cell line and that IL-12p35 mRNA contains a target site for miR-21 in macrophages and DCs, as confirmed by reporter assays, leading to restricted adaptive Th1 responses [105, 107–109]. In contrast, miR-29 suppresses immune responses against intracellular pathogens by targeting IFN-*γ* [110].

Stability of proteins is very important for their biological function. Increasing evidence indicates that miRNAs, together with RNA-binding proteins (RBPs), regulate the stability of numerous cytokine-encoding mRNAs and/or their translation through the AU-rich elements (AREs) of their 3′-UTR regions. For example, both TNF and IL-10 mRNAs contain long AREs targeted by tristetraprolin (TTP), a key factor in mRNA destabilization downstream of the TLR-signalling pathway [67, 68]. In addition, miR-16 cooperates with TTP to mediate TNF mRNA destabilization [97]. Furthermore, a recent study showed that TNF mRNA is directly degraded by miR-221, miR-579, and miR-125b with TTP, leading to LPS tolerance [40, 101]. Among these miRNAs, miR-221 interacts with TTP to accelerate TNF mRNA decay, whereas miR-579 and miR-125b combine with TTP to block TNF mRNA translation [40, 101]. However, some of these effects could result from the upregulation of miR-125b expression [40].

Conversely, miRNAs can also compete with RBPs to protect cytokine-encoding mRNAs from destabilization. For example, miR-466l competes with TTP to bind to the canonical ARE “AUUUA” sequence in IL-10 through its seed region. This binding effect protects the IL-10 mRNA from TTP-mediated degradation [106]. Interestingly, environmental factors are also involved in cytokine-mediated stability of encoding mRNA. For example, under serum starvation, miR-369-3 directly interacts with the ARE in TNF mRNA to initiate TNF mRNA translation, and this interaction depends on the recruitment of the fragile-X mental retardation-related protein 1 and argonaute 2 [102]. In contrast, miR-369-3 represses the expression of TNF when the cells are actively proliferating [42]. It has also been shown that miR-155 is required for TNF mRNA stabilization, as miR-155-deficient B cells and/or miR-155-deficient mice fail to produce increased levels of TNF after LPS injection, although a direct binding site for miR-155 in the TNF mRNA has not yet been identified [40, 42]. In addition to the insights discussed above, further studies are needed to elucidate whether other TLR-responsive cytokines are subject to feedback regulation by miRNAs.

### 3.5. miRNA-Mediated Regulatory TLR-Associated Regulatory Molecules

miRNAs can target TLR-associated regulatory molecules for regulating TLR-signalling pathways (Table 2). A recent study indicated that miR-132 can target acetylcholinesterase (ACHE), a key regulator of TLR-signalling pathways, to increase the acetylcholine-mediated negative regulation of TLR-signalling pathways [49]. Next, miR-21 targets tumour suppressor protein programmed cell death 4 (PDCD4), an inhibitor of eukaryotic translation initiation factor 4F, in macrophages, thus enhancing innate immune responses in the early phase of pathogen infections [63, 111, 112]. In addition, the inhibition of PDCD4 expression by miR-21 increases IL-10 secretion, suggesting complex roles of TLR-induced cytokine production in pathogen infections [63, 111, 112]. Moreover, Src homology 2 domain-containing inositol-5′-phosphatase 1 (SHIP1), a negative regulator of TLR-signalling pathways and inflammatory responses, has been identified as a target of miR-155 [33, 43–45]. The increased expression of miR-155 in response to LPS stimulation or pathogen infection in macrophages accompanies the decreased expression of SHIP1 [32, 43–46, 111, 112]. Furthermore, miR-155 has also been shown to target suppressor of cytokine signalling 1 (SOCS1), which is another negative regulator of TLR-signalling pathways [47, 48, 113]. The expression of miR-155 is also upregulated in macrophages during RNA virus infection, and upregulation of miR-155 provides positive feedback regulation to TLR3- and TLR4-triggered antiviral innate immune responses by promoting type I IFNs signalling via targeting of SOCS1 [47, 113]. However, IL-10 inhibition of miR-155 expression increases SHIP1 and SOCS1 expression and mitigates TLR signalling [47, 48, 62]. Overall, these findings indicate that miR-155 expression is upregulated and inhibits the expression of the negative regulators SHIP1 and SOCS1 in the early phase of TLR4 and endosomal RNA-sensing TLR activation, allowing TLR signal transduction and cytokine production. Later in the response, the increased miR-21 induces IL-10 production by repressing PDCD4 expression, and IL-10 then feeds back to the pathway to reduce miR-155 expression, thereby increasing SHIP1 and SOCS1 expression and limiting the TLR4 and endosomal RNA-sensing TLR-signalling pathways.

Many other miRNAs also target other regulatory molecules involved in regulating the TLR-signalling pathways (Table 2). Notch1, a known positive regulator of IL-12p70 production in DCs, has been confirmed as a target of miR-146a. miR-146a targeting Notch1 suppresses IL-12p70 production in TLR9-triggered DCs [35]. In addition, miR-148a/b and miR-152 can inhibit the expression of calcium/calmodulin-dependent protein kinase II, and thus they regulate TLR-signalling pathways [114]. Furthermore, miR-181 and miR-17/92 suppress TNF-induced cytokine production in epithelial cells by targeting p300/cyclic AMP response element-binding protein-associated factor that is a coactivator and acetyltransferase that promotes histone acetylation and gene transcription [115, 116]. Cytokine-inducible Src homology 2 (CIS) protein and SOCS4, as the regulatory molecules, have been identified as direct targets of miR-98 and let-7. Furthermore, LPS stimulation and the pathogen (*Cryptosporidium parvum, C. parvum*) infection can induce CIS and SOCS4 expression by downregulating miR-98 and let-7 expression in biliary epithelial cells [75, 117, 118]. These studies suggest that miRNAs may be responsible for coordinately regulating the CIS and SOCS expression in human biliary epithelial cells, but the roles of miRNAs in regulating the CIS and SOCS expression can be changed after* C. parvum* infection. Interestingly, TLR-dependent induction of miR-101 appears to provide positive feedback loop control of innate immune responses through miR-101-mediated suppression of MAPK phosphatase 1, an inhibitory regulator of TLR-signalling pathways [119]. Thus, miRNAs are responsible for regulating regulatory molecules to fine-tune TLR-signalling pathways and downstream events.

## 4. Summary

Over the past decade, significant progress has been made in our understanding of the roles of miRNAs in the immune system. The functions of miRNAs have been investigated in detail, particularly in regulating TLR-signalling pathways and innate immune responses by targeting multiple molecules at multiple levels. Subtle differences in TLR-induced miRNA expression profiles revealed by a number of studies have been found to be closely related to ligands, as well as correlated with treatment time, technology used, and cell types. However, the mechanisms regarding TLR-signalling pathways-mediated miRNA expression regulation, through transcriptional repression or posttranscriptional destabilization, need to be further deeply investigated. Given the multifunctional roles of miRNAs in TLR-signalling pathways, miRNA targeting these pathways appears relatively more effective, economical, and common rather than completely shutting them down by abolishing receptor and cytokines expression.

Importantly, studying the central roles of miRNAs in regulating TLR-signalling pathways indicates that TLR-signalling pathways induce multiple miRNAs, which in turn regulate the strength, location, and timing of the TLR-signalling pathways, and might be involved in controlling the switch from a strong early inflammatory response to the resolution phase of the inflammatory process in a timely and orchestrated manner. The innate immune system utilizes multiple miRNAs to properly regulate its functional capacity, creating a finely tuned balance between activation and repression in TLR-signalling pathways. Interestingly, several miRNAs work together with RBP to regulate TLR-responsive mRNA stability and translation in different ways. These findings open up an exciting new area in the regulation of TLR-signalling pathways.

Besides the TLR-signalling pathways, some miRNAs, such as miR-146a, miR-466l, miR-24, miR-122, miR-378, miR-30e, miR-29a, and miR-223, are induced by signalling pathways of retinoic acid-inducible gene-I- (RIG-I-) like receptors and/or nucleotide-binding oligomerization domain- (NOD-) like receptors. Furthermore, they use feedback mechanisms to regulate these signalling pathways and downstream events [26, 56, 98, 99, 130–135]. However, the roles of miRNA-mediated regulation are only beginning to emerge. A wide variety of pathogens, especially DNA viruses, express the highest number of miRNAs, and then these miRNAs directly modulate PRR-signalling pathways and innate immune responses to establish a cellular environment conducive for viral infection and replication [134, 136–141]. More strikingly, a number of studies have revealed that several miRNAs, such as miR-21, miR-29a, and let-7b, can even serve as physiological ligands of the single-stranded RNA-sensing TLRs [142–146]. However, the precise regulatory roles of miRNAs in PRR-mediated signalling pathways and innate immune responses are still not fully understood, especially how these miRNA networks interact to optimize PRR-signalling pathways and inflammatory responses. Moreover, as newly identified regulators, the mechanisms by which miRNAs, in combination with other regulatory mechanisms, control the outcome of immune responses need to be elucidated in future studies. Understanding the roles of miRNAs in regulating PRR-signalling pathways, especially TLR-signalling pathways, innate immune responses, and viral immune evasion may provide important clues for identifying novel and attractive drug targets to inflammatory diseases, cancer, autoimmunity, and infections.

## Figures and Tables

**Table 1:** miRNAs regulated by TLRs.

miRNA	TLRs	Signalling molecules	Transcription factors/cofactors	Cell types	Stimulus	Reference
Upregulated						
miR-146a	TLR2, TLR3, TLR4, TLR5	MyD88	NF-*κ*B (P65)	THP1 cells, BMDMs, T cells, neural cells, MΦ, DCs, epithelial cells, PMN	LPS, TNF-*α*, IL-1*β*, LMP1, H_2_O_2_, VSV, RIG-I	[19–35]
miR-155	TLR2, TLR3, TLR4, TLR9	MyD88, TRIF, KSRP	AP-1 (Fos/Jun), NF-*κ*B (P65/P50)	BMDMs, THP1 cells, DCs, monocytes, MΦ, Treg cells	LPS, LMP1, TNF-*α*, KSHV, *H. pylori*, IFN-*β*, oxidized LDL, alcohol	[19, 28, 30, 33, 34, 36–48]
miR-132	TLR2, TLR4, TLR9	ND	cREB	THP1 cells, splenocytes, MΦ, monocytes, BMDMs,	KSHV	[19, 49–51]
miR-223	TLR4	ND	ND	Inflamed lung tissue, DCs	ND	[19, 38, 52–56]
miR-147	TLR2, TLR3, TLR4	MyD88, TRIF	NF-*κ*B, IRF3	BMDMs, RAW264.7 cells, THP1 cells, alveolar MΦ	ND	[57]
miR-9	TLR2, TLR4, TLR7, TLR8	MyD88	NF-*κ*B	Monocytes, granulocytes, MΦ, PMN, biliary epithelial cells	LPS, TNF-*α*, IL-1*β*	[10, 58]
miR-27b	TLR4	ND	NF-*κ*B (P65)	MΦ	LPS, *C. parvum *	[59–61]
let-7e	TLR4	AKT1	NF-*κ*B	Peritoneal MΦ	ND	[62]
miR-21	TLR4	MyD88, TRIF	NF-*κ*B (P65/P50) AP-1 (Fos/Jun)	RAW264.7 cells, BMDMs, B cells, PBMCs, H69 cholangiocytes, biliary epithelial cells, gastric and breast cancer cell line, promyelocytic leukemia cell line	LPS, LMP1, nicotine, *C. parvum*, PMA; ectopic expression of HER2/neu	[23, 52, 60, 61, 63–68]
miR-125b	TLR4	ND	ND	Biliary epithelial cells, LPS-tolerized THP1 cells, fibroblast-like synoviocytes, H69 cholangiocytes	LPS, *C. parvum *	[61, 69]
miR-16	ND	ND	NF-*κ*B (P65/P50)	Gastric cancer cells	Nicotine	[10, 64]
miR-23b	ND	ND	NF-*κ*B (P65)	Biliary epithelial cells	LPS, *C. parvum *	[10, 60, 61]
miR-30b	ND	ND	NF-*κ*B (P65)	Biliary epithelial cells	LPS, *C. parvum *	[10, 60, 61]
miR-301a	ND	ND	NF-*κ*B (P65/P50)	Pancreatic cancer cells	TNF-*α*	[10, 70]
miR-212	ND	ND			KSHV, EtOH	[50]
miR-146b	ND	ND	AP-1 (Fos)	Glioblastoma cells, ovarian cancer cells	Platelet-derived growth factor	[71]
Downregulated						
let-7i	TLR4	ND	NF-*κ*B, C/EBP*β*	H69 cholangiocytes	LPS, *C. parvum *	[72–75]
miR-98	TLR4	ND		H69 cholangiocytes	*C. parvum *	[75]
miR-125b	TLR4	AKT1	NF-*κ*B	Splenocytes, BMDMs, DCs, RAW264.7 cells, macrophages	ND	[38, 40, 62]
miR-29b	ND	ND	NF-*κ*B (P65), YY1, SP1, HDAC3, HDAC1	Myoblast cell, acute myeloid leukemia cells	TNF-*α*	[76, 77]

MyD88: myeloid differentiation primary-response protein 88; BMDM: bone marrow-derived macrophage; PMN: polymorphonuclear neutrophil; LMP1: latent membrane protein 1; RIG-I: retinoic acid-inducible gene-I; VSV: vesicular stomatitis virus; TRIF: TIR domain-containing adaptor protein inducing IFN-*β* (also known as TICAM1, the TIR domain-containing adapter molecule 1); KSRP: KH-type splicing regulatory protein; JNK: JUN N-terminal kinase; C/EBP*β*: CCAAT/enhancer-binding protein-*β*; KSHV: Kaposi's sarcoma herpesvirus; LDL: low-density lipoprotein; Treg cell: regulatory T cell; *C. parvum*: *Cryptosporidium parvum*; YY1: Yin Yang 1; SP1: specificity protein 1; HDAC3: histone deacetylase 3; HDAC1: histone deacetylase 1; ND: not determined.

**Table 2:** Verified targets of miRNAs in TLR-signalling pathway.

Target mRNA	miRNA(s)	Reference
Receptors		
TLR4	let-7i, let-7e, miR-223, miR-146a, miR-146b, miR-511	[25, 53, 62, 72, 78, 79]
TLR3	miR-223, miR-26a	[53, 80]
TLR2	miR-105, miR-19a/b, miR-143, miR-146a	[81–84]
Signalling proteins		
IRAK1	miR-146a, miR-146b, miR-21	[19, 26, 28, 31, 85]
TRAF6	miR-146a, miR-146b	[19, 26, 79]
IRAK2	miR-146a	[19, 26]
MyD88	miR-146b, miR-155, miR-200b, miR-200c, miR-21, miR-149, miR-203	[29, 30, 54, 79, 86–88]
IRAK4	miR-146a, miR-132, miR-212	[51]
TRAF4	miR-29	[89]
FADD	miR-155	[38, 40]
IKK*β*	miR-155, miR-199a	[38, 40, 54, 88]
IKK*ε*	miR-155	[38, 40]
TAB2	miR-155	[38, 40]
RIPK1	miR-155	[38, 40]
TIRAP	miR-145	[31]
BTK	miR-348	[69, 90]
IKK*α*	miR-223	[91]
MKK4	miR-92a	[92]
STING	miR-24	[56]
ISGs	miR-132	[50]
Transcription factors		
P38 MAPK	miR-155	[38]
NF-*κ*B1	miR-9, miR-210	[58, 93]
NF-*κ*Bp65	miR-329	[94]
STAT3	miR-17-5p, miR-20a, miR-223	[55, 95]
C/EBP*β*	miR-155	[32, 33]
PPAR*γ*	miR-27b	[59]
p300	miR-132	[50]
FOXP3	miR-155	[34]
Ets2	miR-155	[96]
Functional cytokines		
IL-8	miR-146a, miR-16	[22, 97]
RANTES	miR-146a	[22]
IFN-*α*	miR-466l	[98]
IFN-*β*	let-7b, miR-26a, miR-34a, miR-145	[11, 99]
TNF	miR-125b, miR-187, miR-16, miR-221, miR-579, miR-369-3, miR-155	[40, 42, 67, 97, 100–102]
IL-6	miR-16, miR-365, miR-142-3p, miR-187	[11, 73, 103, 104]
IL-10	miR-106a, miR-106b, miR-466l	[105, 106]
IL-12p35	miR-21	[107–109]
IFN-*γ*	miR-9, miR-21	[108, 110]
IL-12p40	miR-187, miR-21	[108, 109]
Regulators molecules		
ACHE	miR-132	[49]
PDCD4	miR-21	[63, 111, 112]
SHIP1	miR-155	[32, 33, 43–46, 62, 111, 112]
SOCS1	miR-155	[47, 48, 62, 113]
Notch1	miR-146a	[35]
CaMKIIalpha	miR-148a/b, miR-152	[114]
PCAF	miR-181, miR-17/92	[115, 116]
CIS	miR-98, let-7	[75, 117, 118]
SOCS4	miR-98, let-7	[75, 117, 118]
SOCS3	miR-203	[118]
MKP-1	miR-101	[119]

IRAK: IL-1R-associated kinase; TRAF6: TNFR-associated factor 6; MyD88: myeloid differentiation primary-response protein 88; FADD: Fas-associated death domain protein; IKK: inhibitor of NF-*κ*B kinase; TAB2: TAK1-binding protein 2; RIPK1: receptor TNFR-interacting serine-threonine kinase 1; TIRAP: TIR domain-containing adaptor protein (also known as MAL, MyD88 adaptor-like protein); BTK: Bruton's tyrosine kinase; MKK4: mitogen-activated protein kinase kinase 4; STAT3: transcription factor signal transducer and activator of transcription 3; C/EBP*β*: CCAAT/enhancer-binding protein-*β*; PPAR*γ*: peroxisome proliferator-activated receptor-*γ*; RANTES: regulated upon activation normal T cell expressed and presumably secreted; FOXP3: forkhead box P3; Ets2: E26 transformation-specific sequence 2; STING: stimulator of interferon genes; ACHE: acetylcholinesterase; PDCD4: programmed cell death 4; SHIP1: Src homology 2 (SH2) domain-containing inositol-5′-phosphatase 1; SOCS: suppressor of cytokine signalling; CIS: cytokine-inducible Src homology 2; CaMKIIalpha: calcium/calmodulin-dependent protein kinase II; PCAF: protein-associated factor; MKP-1: MAPK phosphatase-1.
